# Retraction

**DOI:** 10.1002/cam4.6714

**Published:** 2023-12-18

## Abstract

**‘**Circ‐SPECC1 Modulates TGFβ2 and autophagy under oxidative stress by sponging miR‐33a to promote hepatocellular carcinoma tumorigenesis’ by Bin Zhang, Zhiyi Liu, Kuan Cao, Wengang Shan, Jin Liu, Quan Wen, Renhao Wang, Cancer Med 2020, 9: 5999–6008. The above article, published online on 6 July 2020 in Wiley Online Library (https://onlinelibrary.wiley.com/doi/full/10.1002/cam4.3219) has been retracted by agreement between journal's Editor in Chief, Stephen Tait, and John Wiley & Sons Ltd.

The retraction has been agreed following an investigation based on allegations raised by third parties. Multiple image elements in Figures 1, 2, and 7 were found to have been published elsewhere in a different scientific context. Thus, the editors consider the conclusions of this article to be invalid. The authors did not respond when asked to collaborate during the investigation and confirm the retraction.

**‘**Circ‐SPECC1 Modulates TGFβ2 and autophagy under oxidative stress by sponging miR‐33a to promote hepatocellular carcinoma tumorigenesis’ by Bin Zhang, Zhiyi Liu, Kuan Cao, Wengang Shan, Jin Liu, Quan Wen, Renhao Wang, Cancer Med 2020, 9: 5999–6008. The above article, published online on 6 July 2020 in Wiley Online Library (https://onlinelibrary.wiley.com/doi/full/10.1002/cam4.3219) has been retracted by agreement between journal's Editor in Chief, Stephen Tait, and John Wiley & Sons Ltd.

The retraction has been agreed following an investigation based on allegations raised by third parties. Multiple image elements in Figures 1, 2, and 7 were found to have been published elsewhere in a different scientific context. Thus, the editors consider the conclusions of this article to be invalid. The authors did not respond when asked to collaborate during the investigation and confirm the retraction.

